# Spin–valley locking in the normal state of a transition-metal dichalcogenide superconductor

**DOI:** 10.1038/ncomms11711

**Published:** 2016-05-23

**Authors:** L. Bawden, S. P. Cooil, F. Mazzola, J. M. Riley, L. J. Collins-McIntyre, V. Sunko, K. W. B. Hunvik, M. Leandersson, C. M. Polley, T. Balasubramanian, T. K. Kim, M. Hoesch, J. W. Wells, G. Balakrishnan, M. S. Bahramy, P. D. C. King

**Affiliations:** 1 SUPA, School of Physics and Astronomy, University of St Andrews, St Andrews, Fife KY16 9SS, UK; 2 Department of Physics, Norwegian University of Science and Technology (NTNU), N-7491 Trondheim, Norway; 3 Diamond Light Source, Harwell Campus, Didcot, OX11 0DE, UK; 4 Max Planck Institute for Chemical Physics of Solids, Nöthnitzer Straße 40, 01217 Dresden, Germany; 5 MAX IV Laboratory, Lund University, PO Box 118, 221 00 Lund, Sweden; 6 Department of Physics, University of Warwick, Coventry CV4 7AL, UK; 7 Quantum-Phase Electronics Center and Department of Applied Physics, The University of Tokyo, Tokyo 113-8656, Japan; 8 RIKEN center for Emergent Matter Science (CEMS), Wako 351-0198, Japan

## Abstract

Metallic transition-metal dichalcogenides (TMDCs) are benchmark systems for studying and controlling intertwined electronic orders in solids, with superconductivity developing from a charge-density wave state. The interplay between such phases is thought to play a critical role in the unconventional superconductivity of cuprates, Fe-based and heavy-fermion systems, yet even for the more moderately-correlated TMDCs, their nature and origins have proved controversial. Here, we study a prototypical example, 2H-NbSe_2_, by spin- and angle-resolved photoemission and first-principles theory. We find that the normal state, from which its hallmark collective phases emerge, is characterized by quasiparticles whose spin is locked to their valley pseudospin. This results from a combination of strong spin–orbit interactions and local inversion symmetry breaking, while interlayer coupling further drives a rich three-dimensional momentum dependence of the underlying Fermi-surface spin texture. These findings necessitate a re-investigation of the nature of charge order and superconducting pairing in NbSe_2_ and related TMDCs.

## Abstract (web summary)

The origin of intertwined electronic orders in transition-metal dichalcogenides has long been debated. Here, Bawden *et al*. report that the normal state, from which these phases emerge, is unexpectedly spin-polarized, with spins locked to both valley and layer pseudospins.

## Graphical abstract

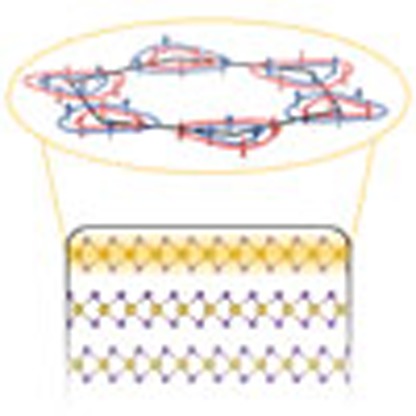

In combination with broken structural inversion symmetry, spin–orbit coupling (SOC) provides a powerful route to stabilize spin-polarized electronic states without magnetism. This can give rise to electrically-tuneable spin splittings via the Rashba effect, promising new technological developments in spintronics^1^, and underpins the formation of spin-helical Dirac cones at the surfaces of topological insulators^2^. It is strongly desired to realize similar effects in systems where more pronounced electronic interactions drive the emergence of collective phases. In non-centrosymmetric superconductors, for example, spin-splitting driven by strong SOC is expected to induce a mixing of spin-triplet and singlet superconducting order parameters^3^, and offers potential for stabilizing topological superconductors^4^. Yet, identifying suitable candidate materials has proved a major challenge to date. Partly this is driven by a relative dearth of non-centrosymmetric metals, while for non-magnetic systems in which the centre of inversion is maintained, robust-spin degeneracies of their electronic states would typically be expected due to the dual constraints of time-reversal and inversion symmetry.

In contrast, we show here from spin- and angle-resolved photoemission (ARPES) measurements that the normal state of the centrosymmetric TMDC superconductor 2H-NbSe_2_ (hereafter denoted NbSe_2_) hosts a strong layer-resolved and momentum-dependent spin polarization of its electronic states at and in the vicinity of the Fermi level. We attribute this as a consequence of a recently-realized form of spin polarization that can emerge in globally centrosymmetric materials in which constituent structural units nonetheless break inversion symmetry^5^,^6^,^7^. Together with first-principles calculations, we show how this drives a critical and complex interplay of interlayer interactions and SOC in NbSe_2_. This yields a rich underlying spin-polarized electronic landscape from which charge order and superconductivity emerge upon cooling.

## Results

### Bulk electronic properties of NbSe_2_

**Figure 1:**
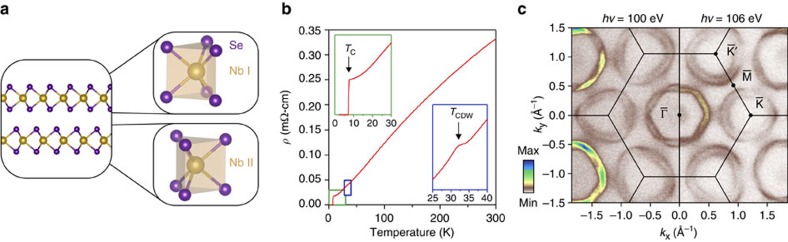
Superconductivity and charge-density wave order in 2H-NbSe_2_. (**a**) Centrosymmetric bulk crystal structure (side view) of 2H-NbSe_2_. This is formed by stacking non-centrosymmetric layers of *D*_3*h*_ symmetry with 180° relative rotations, restoring the bulk inversion centre. (**b**) Resistivity measurements show clear signatures of charge-density wave formation at *T*_CDW_≈33 K and superconductivity at *T*_c_≈7 K (magnified in the insets). (**c**) Normal-state Fermi surface measured by ARPES with *hν*=100 eV (left-hand-side) and *hν*=106 eV (right-hand-side); *E*_*F*_±20 meV. This consists of two Nb-derived barrels centred around the zone-corner 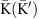 points, two Nb-derived barrels at the Brillouin zone centre and an additional central diffuse pocket (most visible at *hν*=106 eV) predominantly derived from Se *p*_*z*_ orbitals.

We consider here exclusively the layered 2H polymorph (Fig. 1a). Each layer forms a graphene-like honeycomb structure with Nb occupying the A sublattice and two Se atoms situated on the B sublattice. These lie out of the basel plane, equidistant above and below the transition metal. The unit cell contains two such layers, stacked along the *c*-axis with 180° in-plane rotation. Our resistivity measurements (Fig. 1b) from single-crystal NbSe_2_ samples show a metallic temperature-dependence. They additionally exhibit a pronounced hump at a temperature of *T*_CDW_≈33 K indicative of charge-density wave (CDW) formation^8^, as well as a sharp superconducting transition at *T*_c_≈7 K. The corresponding normal-state Fermi surface is shown in Fig. 1c, as measured by ARPES. There are two barrels centred around each zone-corner 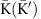 point, which are strongly trigonally-warped. Two further barrels are centred at the 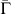 point, the inner of which is hexagonal, while the outer exhibits additional warping. From our first-principles density-functional theory (DFT) calculations, and consistent with previous studies^9^,^10^,^11^, we assign all four of these Fermi surface sheets as being predominantly derived from Nb 4*d* orbitals. Additional spectral weight at the zone centre is evident in our ARPES measurements for selected photon energies, which we attribute as a fifth, highly three-dimensional, Fermi surface sheet of predominantly Se *p*_*z*_ orbital character.

**Figure 2:**
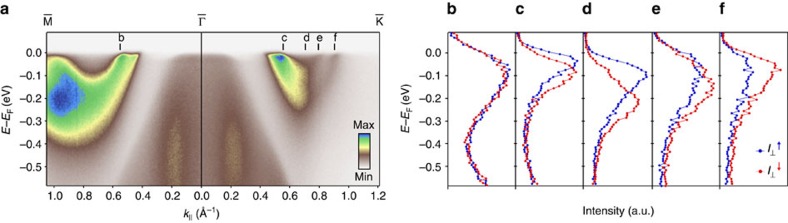
Spin-polarized bulk electronic structure. (**a**) Dispersion measured by ARPES (*hν*=22 eV) along the 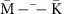 direction. (**b**–**f**) Spin-resolved EDCs at the momenta marked in **a**, revealing a strong spin polarization of these electronic states along 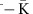. The horizontal axis tick marks denote the zero of each EDC.

This sheet also contributes diffuse filled-in intensity, due to the finite out-of-plane momentum (*k*_*z*_) resolution of ARPES, close to the zone centre in measurements of the electronic dispersions along in-plane high-symmetry directions (Fig. 2a). The Nb-dominated states, on the other hand, yield clear spectral features close to their Fermi crossings. Kinks in their measured dispersion and a decrease in linewidth near the Fermi-level point to relatively strong electron–phonon coupling in this system^11^,^12^,^13^. It is these Nb-derived states that are known to host the largest energy gaps at the Fermi level arising from the CDW and superconducting instabilities in this system^13^,^14^,^15^,^16^, although the origins of these have proved highly controversial^8^,^10^,^11^,^13^,^14^,^15^,^16^,^17^,^18^,^19^,^20^,^21^,^22^. To the best of our knowledge, all prior theoretical treatments assume these orders emerge from an electronic liquid of trivially spin-degenerate character. Indeed, standard expectations of group theory would say this must be the case for the centrosymmetric space group (P6_3_/mmc) of 2H-NbSe_2_.

In contrast, we directly observe pronounced spin polarizations of the underlying electronic states in spin-resolved energy distribution curves (spin-EDCs, see Methods) measured along the 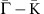 direction, shown in Fig. 2c–f. Close to the saddle point of these bands (Fig. 2d), two clearly-separated peaks can be observed in spin-EDCs. The measured spin polarization is almost entirely out-of-plane (Fig. 2d and Supplementary Fig. 1), with a sign that reverses between the two bands. We attribute this as arising from local inversion symmetry breaking within the individual layers that make up the bulk crystal structure^5^,^6^,^23^. For sufficiently weak interlayer interactions (a point we return to below), pronounced SOC characteristic of the 4*d* transition metal can lift the spin degeneracy of the states localized within each layer, that thus strongly feel the local inversion asymmetry. A layer-dependent sign change of this spin polarization, mediated by the rotated-layer stacking of the bulk crystal structure, would act to restore overall spin degeneracy as required by global inversion and time-reversal symmetries. Nonetheless, the electronic states retain strong layer-resolved spin polarizations. Depth-averaging probes would largely be insensitive to these, but photoemission is a surface sensitive technique. Incoherent superposition of photoelectrons emitted from neighbouring layers of the unit cell would lead to some suppression of the measured spin polarization, while interference effects can further complicate this picture^5^. Nonetheless, the extreme surface sensitivity at the photon energies used here, with an inelastic mean free path on the order of the interlayer separation, renders us predominantly sensitive to the spin texture of the top-most layer of the unit cell.

### Spin–valley locking

**Figure 3:**
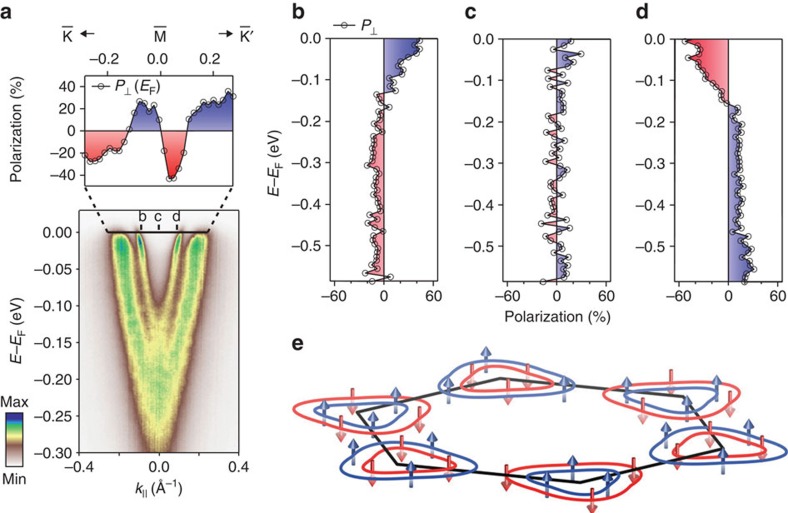
Spin–valley locked Fermi surfaces. (**a**) Dispersion measured (*hν*=22 eV) along the 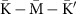 direction, together with the corresponding spin polarisation of a momentum distribution curve at the Fermi level and (**b**–**d**) EDCs at the momenta marked in **a**. These reveal how the sign of the spin polarization for each zone-corner Fermi surface sheet becomes locked to the valley degree of freedom, as shown schematically in **e**.

Our measurements show how such spin textures in NbSe_2_ persist up to the Fermi level. This is evident in spin-resolved EDCs along the 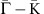 direction (Fig. 2c–f) as well as the spin polarization of EDCs and a Fermi-level momentum-distribution curve along the 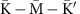 direction (Fig. 3). The latter clearly reveals how the spin polarization reverses sign for each pair of Fermi surface sheets centred on neighbouring 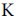 and 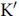 points. This is a natural consequence of time-reversal symmetry. Here, this results in a coupling of the spin to the so-called valley index, the quantum number which distinguishes 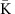
- and 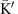-centred Fermi surfaces in NbSe_2_ (Fig. 3e). Such spin–valley coupling has recently been extensively investigated for the band extrema in monolayers of semiconducting TMDCs^24^,^25^,^26^, where it has not only been shown to lead to new physics, such as a valley Hall effect^27^, but also to offer potential for devices exploiting the valley pseudospin^28^,^29^. Our observations here point to a pronounced role of spin–valley coupling also for the low-energy quasiparticle excitations of the metallic 2H-structured TMDCs.

Critically, it is from this spin-polarized Fermi sea that electron–hole and electron–electron pairing interactions drive the formation of CDW order and superconductivity. The largest CDW gaps in NbSe_2_ are located on the zone-corner spin–valley-locked Fermi surfaces^13^. The CDW wave vector is entirely in-plane^30^, making the hidden layer-dependent spin polarizations relevant. The underlying spin textures can also be expected to have important implications for superconductivity. Recent measurements on electrically gated MoS_2_, which is known to host a strong spin–valley locking in its band structure^25^,^26^, have found evidence for unconventional so-called Ising superconductivity^31^,^32^. In this, the pronounced spin–orbit field that induces the underlying spin texture of the electronic states pins the spin of Cooper pair electrons in the out-of-plane direction. This leads to upper critical fields dramatically exceeding the Pauli paramagnetic limit for a magnetic field applied within the *ab*-plane. Similar phenomenology has recently been reported for mono- and few-layer NbSe_2_ (ref. 33), entirely consistent with our direct observation of spin–valley locking in this compound.

In bulk NbSe_2_, the spin-layer locking, and also multi-band nature as compared to gated MoS_2_, raises further prospects for stabilizing a delicate balance between different pairing states. In the normal state, we find that the 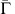-centred Fermi surface barrels, which are known to support a modulated superconducting gap in the bulk^13^,^34^, are also strongly spin-polarized along the 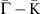 direction (Fig. 2c). Their spin polarization, however, is completely suppressed along 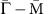 (Fig. 2b). Intriguingly, the largest superconducting gaps of this Fermi surface determined in Rahn *et al*.^13^ are located at the regions of strongest spin polarization evident here. Pronounced superconducting gaps are also known to occur on the zone-corner Fermi surfaces^13^,^15^,^34^, which as demonstrated above, host strong spin–valley locking even in the bulk. Given the pronounced influence of SOC, a mixing of spin-triplet and spin-singlet order parameters could potentially be expected^3^. Forming even pseudospin-singlet Cooper pairs from the strongly spin-polarized Fermi surfaces could necessitate a phase locking between the order parameter of neighbouring Fermi surface sheets. Moreover, the *c*-axis coherence length in NbSe_2_ is much greater than the interlayer separation^35^,^36^. This raises the tantalizing prospect that the inherent coupling between the spin and layer pseudospins reported here could be tuned to drive an instability to an odd-parity pair density-wave state, where the sign of the superconducting gap becomes tied to the layer index^7^,^37^. The proximity of bulk NbSe_2_ and similar compounds to such phases requires further theoretical exploration, and will depend sensitively on the relative importance of inter- and intra-band as well as interlayer pairing interactions.

### Three-dimensional spin structure

**Figure 4:**
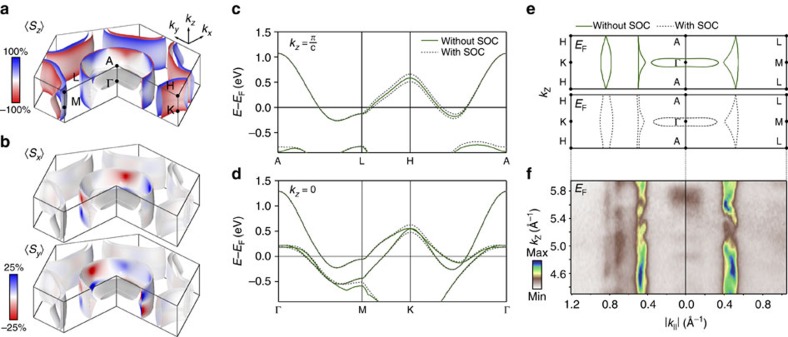
Interplay of interlayer interactions and intralayer inversion symmetry breaking. (**a**,**b**) DFT calculations of the (**a**) out-of-plane and (**b**) in-plane spin polarization of the three-dimensional Fermi surface of NbSe_2_ projected onto the first layer of the unit cell. (**c**,**d**) Calculated electronic structure along (**c**) A-L-H-A (*k*_*z*_=*π*/*c*) and (**d**) Γ-M-K-Γ (*k*_*z*_=0) with and without SOC. (**e**) Corresponding influence of SOC on the Fermi surface contours in the Γ-K-H-A and Γ-M-L-A planes (shown throughout the full three-dimensional Brillouin zone in Supplementary Fig. 2). (**f**) Our experimental ARPES measurements of such *k*_*z*_-dependent Fermi surfaces (*hν*=60–130 eV) are in good general agreement with the theoretical calculations including SOC, supporting a SOC-mediated suppression of interlayer hopping in the Γ-K-H-A plane.

Our DFT calculations (Fig. 4) already reveal a key role of interlayer interactions in mediating and controlling the underlying spin texture of the normal-state bulk Fermi surface. The calculated spin polarization projected onto the first layer of the unit cell is shown throughout the full three-dimensional Brillouin zone in Fig. 4a,b. The momentum-dependent spin polarizations are determined by the effective spin–orbit field, **B**_**so**_=*β*(∇**V** × **k**), where *β* is a momentum-dependent scaling factor, ∇**V** is the net electrostatic potential gradient and **k** is the crystal momentum. The horizontal mirror symmetry of each NbSe_2_ structural unit about the transition-metal plane (*σ*_h_ of the D_3*h*_ point group; Fig. 1a) ensures that ∇**V** is entirely within the *xy* plane. Due to the 180° relative rotation of neighbouring NbSe_2_ monolayers in the bulk crystal structure, ∇**V**, and thus **B**_**so**_, has opposite sign for successive layers in the unit cell. This causes the sign of the spin polarization to reverse at each momentum-space point when projected onto layer 2 versus layer 1 of the unit cell (Supplementary Fig. 3). This confirms that local inversion asymmetry within each NbSe_2_ layer drives the formation of the spin-polarized states observed here.

For electronic states whose wavefunctions are completely delocalized over both layers of the unit cell, the spin–orbit field from each layer cancels. Such states are consequently unpolarized, restoring the conventional expectations for a centrosymmetric space group. This can be observed for the highly three-dimensional pancake-like Fermi surface at the Brillouin zone centre here. In contrast, for electronic states predominantly localized within individual layers of the unit cell, strong layer-dependent spin–orbit fields mediate large layer-resolved spin polarizations. This is ideally realized at the three-dimensional Brillouin zone boundary along *k*_*z*_ (*k*_*z*_=*π*/*c*). In a tight-binding picture, for this *k*_*z*_, interlayer hoppings within a unit cell and between neighbouring unit cells are out of phase with each other and thus cancel^10^. This can be directly visualized by comparing the dispersion of the electronic states along the Γ-M-K-Γ and A-L-H-A directions: neglecting SOC, a single fourfold degenerate band crosses the Fermi level in the *k*_*z*_=*π*/*c* plane (Fig. 4c), whereas this is split into a pair of twofold degenerate bands by interlayer interactions for |*k*_*z*_|<*π*/*c*, as evident in Fig. 4d.

With its forbidden interlayer coupling, the electronic structure for the *k*_*z*_=*π*/*c* plane is thus formally equivalent to that of an isolated monolayer. The spin–orbit field is consequently maximized, driving the largest (>90%) layer-dependent spin-polarizations. These are purely out-of-plane (Fig. 4a,b), as (∇**V** × **k**) must lie entirely along *z* at the Brillouin zone boundary along *k*_*z*_. Along the A-L direction, however, a vertical mirror plane in the crystal structure forbids any out-of-plane component of (∇**V** × **k**). The spin–orbit field must therefore be strictly zero along A-L. This enforces a touching, and hence spin degeneracy, of the zone-centre Fermi surface barrels along this direction, which are otherwise strongly spin-polarized throughout the *k*_*z*_=*π*/*c* plane.

For other *k*_*z*_ through the Brillouin zone, the spin–orbit field strength can be partially suppressed by finite interlayer coupling. Moreover, the *z*-component of the momentum induces a non-zero component of (∇**V** × **k**) in the *xy* plane. Together, this causes not only the magnitude of the Fermi surface spin polarization, but also its vectorial spin texture, to develop a strong dependence on both the in- and out-of-plane momentum (Fig. 4a,b). Within the Γ-M-L-A plane, the calculated Fermi surface crossings are relatively strongly dispersive in *k*_*z*_ (Fig. 4e). This is consistent with our experimental measurements of the *k*_*z*_-dependent Fermi surface (Fig. 4f) and points to significant interlayer coupling. Even away from *k*_*z*_=*π*/*c*, where there are strict spin degeneracies along A-L as discussed above, the Fermi surface spin polarizations therefore become strongly suppressed in the entire vicinity of the Γ-M-L-A plane.

For the Γ-K-H-A plane, on the other hand, we find that the *k*_*z*_ dispersion of the Fermi surface crossings are significantly reduced by the inclusion of spin–orbit coupling in the calculations (Fig. 4e), in keeping with our experimental measurements of more two-dimensional Fermi contours for this plane (Fig. 4f). This reduction in *k*_*z*_ dispersion is achieved by a lifting, via SOC, of the fourfold degeneracy that would otherwise be present along the A-H line, and is accompanied by the emergence of strong spin polarization of the Fermi surface crossings for *k*_*z*_=*π*/*c*. This is in contrast to the A-L line, where the energetic degeneracy of the Fermi crossings is protected by their symmetry-enforced spin degeneracy. The reduction of *k*_*z*_ dispersion within the Γ-K-H-A plane is equivalent to a spin–orbit-mediated suppression of interlayer hopping here^28^. This allows relatively strong spin polarizations to be maintained in the vicinity of this plane, with only a moderate suppression of the spin–orbit field strength away from *k*_*z*_=*π*/*c*.

For momenta close to the K-H line, the in-plane momentum is always much larger than the out-of-plane component. This causes (∇**V** × **k**) to remain predominately aligned along *z*. The spin polarizations of the zone-corner Fermi surfaces are thus largely out-of-plane throughout the full Brillouin zone (Fig. 4a). For the Nb-derived zone-centre Fermi surface barrels, however, much stronger (up to ∼25%) in-plane components develop (Fig. 4b). The in-plane spin texture is largely radial to the Fermi surface (Supplementary Fig. 4), and switches sign about the *k*_*z*_=0 plane. This indicates a non-zero component of (∇**V** × **k**) within the plane, with a direction that is tied to the sign of *k*_*z*_. It thus confirms that an in-plane component of the spin texture arises due to finite out-of-plane momentum, where no symmetry constraint exists to cancel the in-plane component of the spin–orbit field for low-symmetry momenta with 0<|*k*_*z*_|<*π*/*c*. The existence of such hidden in-plane spin textures is a unique property of the bulk compound. Indeed, the in-plane spin component goes strictly to zero at the Brillouin zone boundary along *k*_*z*_, enforced by symmetry, and recovering a monolayer-like purely out-of-plane spin texture for *k*_*z*_=±*π*/*c*. The bulk system thus hosts a rich intertwining of spin, orbital and layer degrees of freedom, mediating a three-dimensional nature of its spin texture that can be expected to further modulate its pairing interactions^31^.

## Discussion

Taken together, our results show how the combination of interlayer hopping and intralayer inversion symmetry breaking can lead to particularly rich momentum-dependent spin textures of metallic TMDCs. Suppression of Fermi-surface spin polarization away from the Brillouin zone boundary along *k*_*z*_ will be reduced with decreased interlayer interactions. Indeed, upper critical fields, already known to exceed the Pauli limit for bulk NbSe_2_ (ref. 38), are dramatically enhanced in other 2H-TMDC superconductors as a function of increasing interlayer separation^39^. This points to a susceptibility of the bulk systems to Ising superconductivity similar to that recently observed in isolated monolayers^33^. The delicate balance between interlayer hopping and SOC strength in NbSe_2_ makes this an ideal material for understanding, and ultimately controlling the role of layer-dependent spin polarizations on the collective states and phases of transition-metal dichalcogenides.

## Methods

### ARPES

Spin-resolved ARPES measurements were performed at the I3 beamline of MAX IV Laboratory, Sweden, and ARPES measurements were performed using the I05 beamline of Diamond Light Source, UK. Measurements were performed at temperatures of 50–80 K using *p*-polarized synchrotron light. Scienta R4000 hemispherical electron analyzers were utilized for all measurements. This was additionally fitted with a mini-Mott detector scheme for the spin-ARPES measurements, configured to simultaneously probe the out-of-plane and in-plane (along the analyzer slit direction) component of the photoelectron spin^40^. The finite spin-detection efficiency was corrected using a Sherman function of *S*=0.17 (ref. 40), and the spin-resolved EDCs determined according to


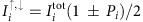


with 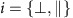, 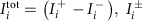 the measured intensity on the individual detectors in the Mott scattering chamber, corrected by a relative detector efficiency calibration, and the total spin polarization,


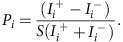


### Calculations

DFT calculations including SOC were performed using the modified Becke–Johnson exchange potential and Perdew–Burke–Ernzerhof correlation functional implemented in the WIEN2K programme^41^. A 20 × 20 × 10 *k*-mesh was employed. The DFT results were downfolded using maximally localized Wannier functions^42^,^43^,^44^, employing Nb 4*d* orbitals and Se 5*p* orbitals as basis functions. The resulting tight-binding Hamiltonian allows a direct extraction of the spin and layer projections of the electronic structure.

### Data availability

Data underpinning this publication can be accessed at http://dx.doi.org/10.17630/edd53357-41e3-4313-9e5e-a0e7780ef4be.

## Additional information

**How to cite this article:** Bawden, L. *et al*. Spin–valley locking in the normal state of a transition-metal dichalcogenide superconductor. *Nat. Commun.* 7:11711 doi: 10.1038/ncomms11711 (2016).

## Supplementary Material

Supplementary Information Supplementary Figures 1-4
