# Supplementary material for: Spin–valley locking in the normal state of a transition-metal dichalcogenide superconductor
Source: Nat Commun. 2016 May 23;7:11711. doi: 10.1038/ncomms11711 (PMC4879244; doi:10.1038/ncomms11711)
Supplement: Supplementary Information — Supplementary Figures 1-4 [file ncomms11711-s1.pdf]

Supplementary Figures

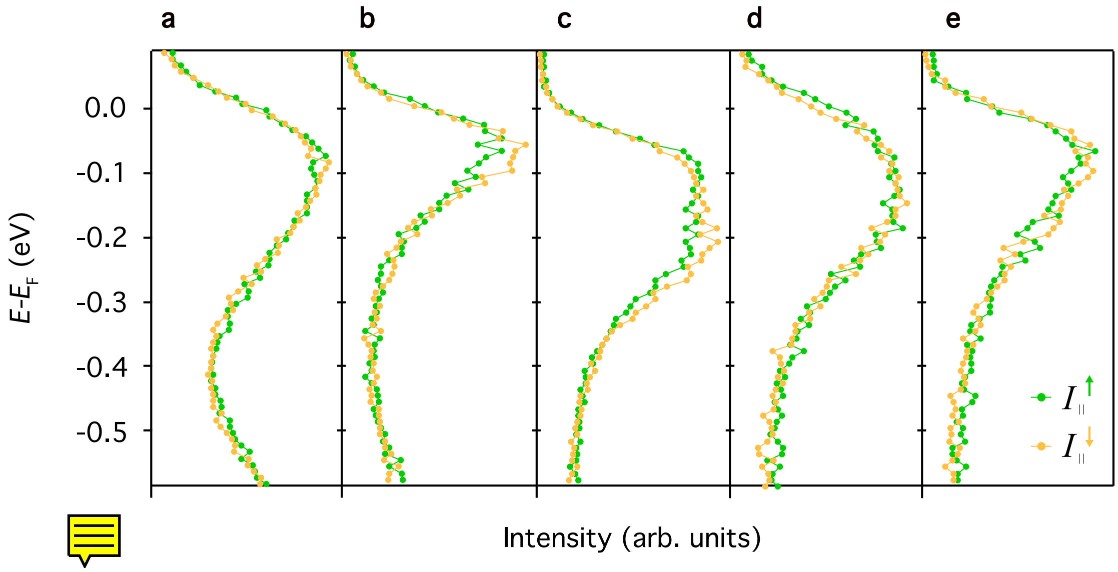

Supplementary Figure 1. **Measured in-plane spin component.** (a-e) Negligible in-plane component of the measured photoelectron spin along the analyser slit (parallel to the $\overline{\Gamma}-\overline{\mathrm{M}}$) direction corresponding to the out-of-plane components shown in (b-f) of Fig. 2 of the main text, respectively. The x-axis tick marks denote the zero of each EDC.

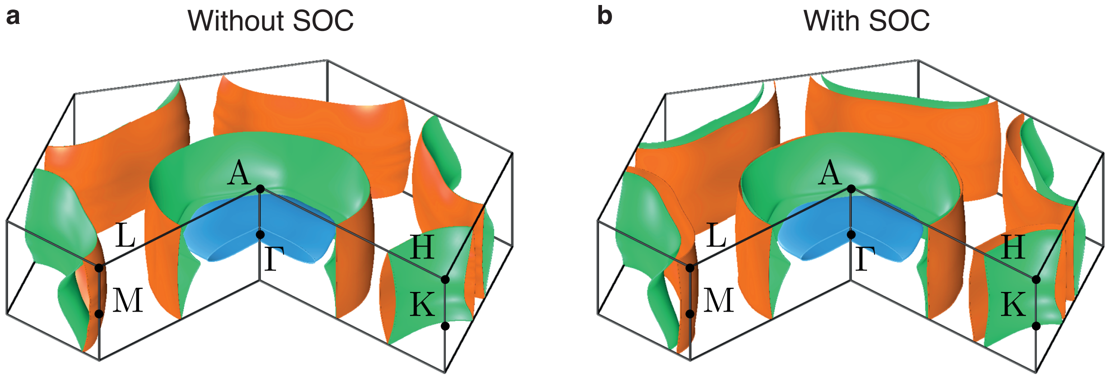

Supplementary Figure 2. **Influence of spin-orbit coupling on the Fermi surface of $NbSe_2$.** (a) Three-dimensional Fermi surface of $NbSe_2$ calculated (a) without and (b) including spin-orbit coupling.

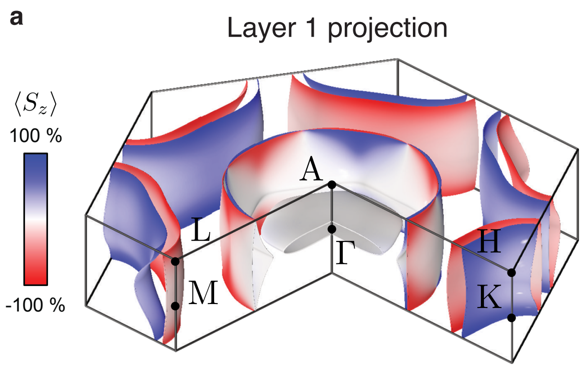

Supplementary Figure 3. **Layer-dependent spin texture.** Calculated out-of-plane component of the Fermi surface spin polarisation projected onto the (a) first and (b) second $NbSe_2$ layer of the unit cell.

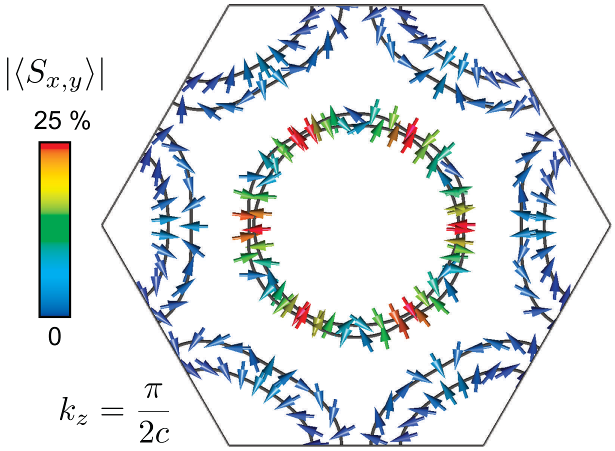

Supplementary Figure 4. **In-plane spin texture.** In-plane vectorial spin texture for the $k_z = \pi/2c$ plane, with the spin direction shown by arrows and the magnitude shown by the colour scaling. This indicates that the in-plane spin configuration is dominantly radial to the Fermi surface.
